# Supervised interprofessional student pain clinic program - efficacy with the utilization of zoom

**DOI:** 10.3389/fpain.2023.1144666

**Published:** 2023-05-23

**Authors:** Brandon Dao, Ling Cao

**Affiliations:** ^1^ College of Osteopathic Medicine, University of New England College of Osteopathic Medicine, Biddeford, ME, United States; ^2^ Biomedical Sciences Department, College of Osteopathic Medicine, University of New England, Biddeford, ME, United States

**Keywords:** zoom, interprofessional education, chronic pain, teamwork, COVID - 19

## Abstract

**Introduction:**

Current medical education curricula in pain management are insufficient to match the prevalence of chronic pain and the needs of patient populations. The Supervised Student Inter-professional Pain Clinic Program (SSIPCP) aims to train healthcare professional students to improve their abilities in chronic pain management in interprofessional (IP) teams. Due to the COVID-19 pandemic, Zoom was employed to allow the program to continue. In this study, survey data from students who participated during and before the COVID-19 pandemic were compared to determine if the program carried out via Zoom can maintain its effectiveness.

**Methods:**

Student pre- and post-program survey data were entered into Microsoft Excel spreadsheet and then graphed and analyzed with Sigma Plot. Surveys assessed knowledge in chronic pain physiology and management, attitude towards IP practice, and perceived team skills in the form of questionnaires and open-ended questions. Paired *t*-tests and Wilcoxon Signed-rank tests were used for two-group comparisons and two-way repeated ANOVA followed by the Holm-Sidak *post-hoc* tests were used for multiple group comparisons.

**Results:**

Overall, students continued to exhibit significant improvement in major areas assessed even with the use of Zoom. Strengths of the programs were also shared across student cohorts regardless of Zoom usage. However, despite their improvements, students who utilized Zoom stated that they would have preferred in-person program activities.

**Conclusion:**

Although students prefer in-person activities, the SSIPCP successfully trained healthcare students in chronic pain management and working in an IP team through Zoom.

## 1. Background

Chronic pain is an ongoing issue that affects millions of U.S citizens. In 2016, the CDC estimated about 50 million adults in the United States experience chronic pain and 19.6 million adults had chronic pain that impacts daily activity (1). Due to the prevalence of chronic pain in the U.S, it costs the nation up to $635 billion each year in the form of medical treatment and lost productivity (2). Although chronic pain continues to be a huge problem, medical school curriculum regarding chronic pain remains lackluster. A study at Johns Hopkins University examining medical schools in North America revealed that pain education is limited and fragmentary (3). Lack of adequate pain education leads to inefficient care for chronic pain patients. A qualitative study reported that medical school students and medical residents felt inadequately prepared to treat chronic pain patients. Without adequate training, students lacked the skills and empathy to treat chronic pain patients effectively (4).

Maine is also no stranger to chronic pain. According to data analysis from Maine All Payer Claims Database (MEAPCD), 29.5% of the total Maine population suffers from chronic pain (5). In the attempt to fill the gap of lack of chronic pain education in Maine, the Supervised Student Interprofessional Chronic Pain Program (SSIPCP) at The University of New England College of Osteopathic Medicine (UNE COM) was created to provide students with the experience of chronic pain patient care in an interprofessional (IP) setting. Students were able to significantly improve their background knowledge regarding chronic pain physiology while improving their ability to work in an IP setting with students from other health care professions (6).

The program has successfully trained students using in-person and on-site settings up until the end of Fall 2019. However, during the acute COVID-19 pandemic, physical distancing measures and state mandates severely limited on-site teaching activities. To combat this, HIPPA-compliant video conferencing utilizing Zoom was employed to allow students to observe the attending physiatrist at the Northern Light Mercy Pain Center in Portland, Maine, perform office visits with chronic pain patients, as well as conduct team meetings. The program in Spring 2020 utilized Zoom for the final team meeting while in Spring 2021 it was completely reliant on Zoom sessions. In this study, survey data from students who participated during and before the COVID-19 pandemic were compared to determine if the program carried out via Zoom could maintain its efficacy.

### 1.1. Zoom as a real time video platform for education

Zoom is a video communication service that was founded in 2011 but has recently gained traction during the COVID-19 pandemic due to its versatility and ease of use. Zoom provides video, voice, and chatting services across all types of electronic devices (7). Within the education sphere, Zoom has been involved in many different school systems supporting traditional, virtual, and hybrid classrooms in the midst of the pandemic. Zoom provides many features, like breakout rooms, screen sharing and annotating to allow for team exercises and presentations. Within medical schools, Zoom has been an important tool as it gave students ease of access to lectures and presentations from home. For students, time that was previously allotted to commuting could be used elsewhere. For hospitals, such as Mercy Hospital, Zoom has been adopted for telehealth appointments during COVID-19 pandemic while protecting patients' privacy. To continue with the SSIPCP during the pandemic, we utilized the Zoom program subscribed by Mercy Hospital. We took advantage of many features of Zoom that contributed to the success of the educational program during COVID-19 pandemic.

## 2. Methods

### 2.1. The supervised student inter-professional pain clinic program (SSIPCP)

The SSIPCP is a 12-week interprofessional training program that recruits students from various health care professions including nursing, osteopathic medicine, occupational therapy, pharmacy, physical therapy, and social work within the University of New England (6). Students were placed into teams with other professions in which they would assess a patient with chronic pain and then create a treatment plan under the supervision of the attending physiatrist. Students have a total of 3 appointments with the patient (students are required to attend at least one appointment due to their class schedules) and 4 team meetings for team discussion. Pre- and post-surveys consisting of questionnaires assessing knowledge in chronic pain physiology and management, attitude and perception towards IP practice, and perceived team skills were conducted. Patient confidentiality and privacy was protected throughout the program. For more details regarding the program, see our previous publication (6).

The SSIPCP began in Spring of 2016 and has been held each semester except that the program in the Fall of 2020 that was cancelled due to COVID-19. The program has successfully trained students using in-person and on-site settings up until Fall 2019. Due to the COVID-19 pandemic, HIPPA-compliant Zoom was employed to allow the program to continue running without physical contact. Participating students in Spring 2020 utilized Zoom for their final team meeting. During patient appointments, the attending physiatrist would be with the patient in the exam room while participating students attended via Zoom. Students in Spring 2021 were completely reliant on Zoom sessions.

The project received IRB exemption from University of New England (protocol#112515-014) and IRB approval from Mercy Hospital (protocol#135).

### 2.2. Outcome measures from the program

Pre- and post-surveys included information about prior interprofessional/chronic pain experiences, KnowPain50 (KP50), Revised Neurophysiology of Pain Questionnaire (RNPQ), Interprofessional Education Perceptions Scale (IEPS), Team Skill Scale (TSS), and open-ended questions. The KP50 quantitatively measured students' knowledge of chronic pain physiology through 50 questions scored out of a total of 250 points. This was a self-assessment tool created to numerically gauge a physician's expertise regarding chronic pain management but can also measure the effectiveness of pain management education programs (8). The RNPQ also quantitatively measured students' knowledge through a true or false survey scored out of 12 points for 12 questions. It was previously used to help patients conceptualize the biological mechanisms of their chronic pain (9). Students' attitude and perception towards IP practice were evaluated using the IEPS, a structurally stable and reliable measurement tool for undergraduate health and social care students (10). The IEPS measures 3 sub items: professional competence and autonomy, perceived need for cooperation, and actual coop for further analysis. Students' perceived abilities to work together in an interdisciplinary setting was measured utilizing the modified TSS (11). In addition, students also assessed their achievement of program learning objectives in the post-survey via a questionnaire using Likert scale (1= Strongly disagree, 2 = Disagree, 3 = Agree, and 4 = Strongly agree, and 0 = N/A).

### 2.3. Statistical analysis

Student de-identified pre- and post-program survey data from Spring 2019, Fall 2019, Spring 2020, and Spring 2021 were entered into Microsoft Excel and then graphed and analyzed with SigmaPlot 10 with Sigma-Stat embedded (Systat Software, Inpixon, Palo Alto, CA). Data from 8 students was removed from data analysis due to missing post-survey data. Paired *t*-tests (when normality tests were passed) and Wilcoxon Signed-rank tests (when normality test did not pass) were used for two-group comparisons and two-way repeated analysis of variance (ANOVA) followed by the Holm-Sidak *post-hoc* tests were used for multiple group comparisons of log transformed data. Data are presented as mean ± SEM. *p *< 0.05 is considered as statistically significant.

### 2.4. Qualitative data analysis

Open ended questions allowed students to evaluate program learning objectives (using Likert scale), along with providing feedback on the strengths and weaknesses of the program. The open-ended feedback questions included were: (1) What did you like best about the training program? (2) What did you like least about the training program? If you could change or improve the training program to address this, what would you do? (3) What about your experience in this training program genuinely surprised you or challenged your previous perceptions both in interprofessional practice and chronic pain management? (4) How do you think this training experience might influence your healthcare practice in the future? Answers for each of these questions were copied and grouped into Microsoft Word to identify the most common themes from students in each participating year. Selected quotes representing major themes are discussed in the results section.

## 3. Results

### 3.1. Participating students and prior experience

There was a total of 54 students from 7 different health care professions who were enrolled into the program from Spring 2019—Spring 2021. Eight students were unable to complete the post-survey but had filled out a pre-survey which was included in the analysis (Table 1).

**Table 1:** Number of students from each health professional program that participated each semester^a^

Programs	# of participants in Spring 2019	# of participants in Fall 2019	# of participants in Spring 2020	# of participants in Spring 2021	Total # of students
Nursing	0	1	0	2	3
Occupational therapy	2	3	1	2	8
Osteopathic medicine	4	3	4	4	15
Pharmacy	2	2	3	1	8
Physical therapy	0	2	3	1	6
Physician assistant	1	0	0	1	2
Social work	3	3	2	4	12
Total # of students	12	14	13	15	54

^a^ Program was cancelled in the fall of 2020; participating students in Spring 2020 utilized Zoom for their final team meeting while students in Spring 2021 were completely reliant on Zoom sessions.

**Figure 1:**
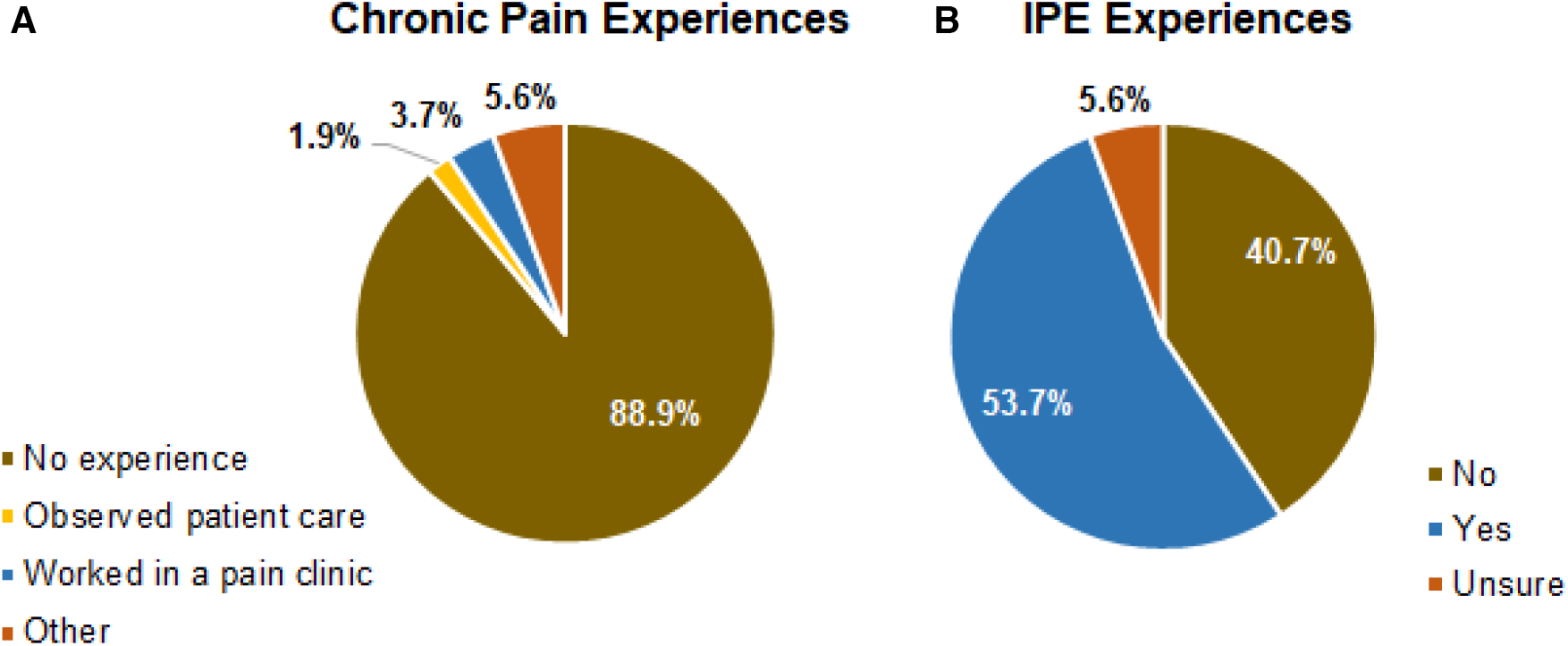
(**A,B**) student participants’ previous chronic pain and IPE experiences. Percentage of students who have or have not had prior experiences working with chronic pain patients and in what form are shown in (**A**). Percentage of students who have or have not worked in an IP setting are shown in (**B**).

In terms of students' prior experience with chronic pain, the majority of participants (48 in 54, 88.9%) had no prior chronic pain-related experiences while the rest either observed chronic pain patient care or worked in a pain clinic (Figure 1A). In regard to interprofessional education experience, a slightly over half of the participants (29 in 54, 53.7%) of the participating students stated they have had prior interprofessional experiences (Figure 1B).

### 3.2. Overall improvement in outcome measurements

**Figure 2:**
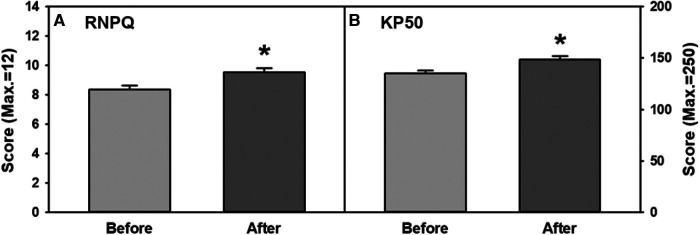
(**A,B**) overall combined improvements on participants’ knowledge in chronic pain physiology and pain management measured with (A) RNPQ and (B) KP50 before and after the program. RNPQ, Revised neurophysiology of pain questionnaire; KP50, KnowPain50.

When students' knowledge in chronic pain physiology and pain management before and after the program was analyzed, there were significant increases in the participants' revised RNPQ score (Figure 2A, Wilcoxon signed-rank test, *p* < 0.001) and KP50 score (Figure 2B, Wilcoxon signed-rank test, *p* < 0.001).

**Figure 3:**
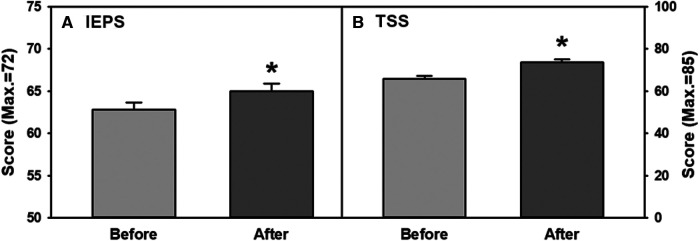
(**A,B**) overall combined improvements of participants’ perception in interprofessional teamwork abilities measured with (**A**) IEPS and (**B**) TSS scores before and after the program. IEPS, Interprofessional education perceptions scale. TSS, Team skill scale.

The perception of participants' attitude and abilities to work in an interprofessional setting significantly improved as is reflected in an increased score in the IEPS questionnaire (Figure 3A, two-tailed paired t-test, *p* < 0.002). Students’ overall perception of their teamwork abilities were significantly increased as well, which is shown by a significant increase in the TSS score (Figure 3B, two-tailed paired t-test, *p* < 0.001).

### 3.3. Improvement in outcome measurements by semester/year

**Figure 4:**
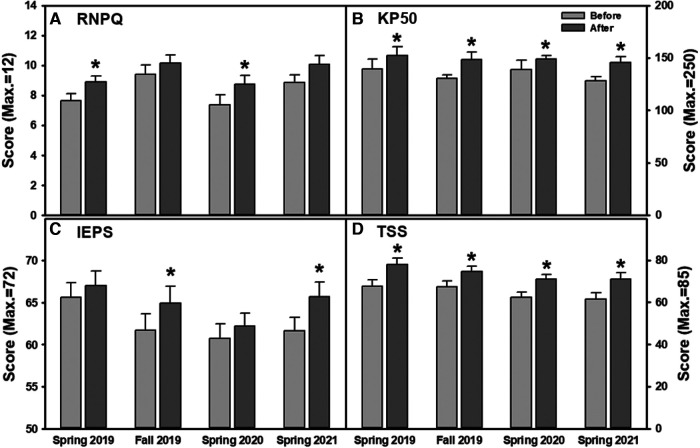
(**A–D**) changes in outcome measures for each program session. Knowledge in chronic pain physiology and pain management were assessed with (**A**) RNPQ and (**B**) KP50. Interprofessional teamwork abilities were measured through (**C**) IEPS and (**D**) TSS.

The improvement regarding knowledge in chronic pain physiology and pain management was compared between each program session. Although the average scores of RNPQ were increased in all semesters, statistical significance was found in Spring 2019 and Spring 2020 sessions (Figure 4A, ANOVA, *p* = 0.036, *p* = 0.019, respectively). Pre vs. Post program KP50 scores revealed significant improvement regardless of the semester/year of participation (Figure 4B, ANOVA, *p* < 0.05 for all). When students' perception and attitude towards interprofessional practice were analyzed, IEPS scores significantly increased in Fall 2019 and Spring 2021 (Figure 4C, ANOVA, *p* = 0.048, *p* = 0.012, respectively) with the overall increase in the average score observed in all sessions. Regarding students' perception of their teamwork abilities, TSS scores were significantly increased in all program sessions (Figure 4D, ANOVA, *p* < 0.05 for all). When comparing between the participating years, there were no significant differences in the extent of student improvement after program completion. Furthermore, when the 3 IEPS sub-items (professional competence and autonomy, perceived need for cooperation, and actual cooperation) were analyzed, there were no notable patterns of significance when comparing extent of improvement between Spring 2019, Fall 2019, Spring 2020, and Spring 2021 (sub-item data not shown). Within the category of professional competence and autonomy, Fall 2019 (ANOVA, mean 25.333 ± 0.748, *p* = 0.038) and Spring 2021 (ANOVA, mean 26.231 ± 0.718, *p* = 0.030) displayed significant improvement. The category of perceived cooperation revealed no significant differences. The third category of actual cooperation showed significant improvement in only Spring 2021 (ANOVA, mean 26.500 ± 0.721, *p* = 0.012).

### 3.4. Learning objective achievement

In the post-survey, students provided feedback on whether they felt they had achieved the learning objectives of the program (Table 2). The results revealed that the program successfully and consistently met al.l learning objectives in each program session. A total of 7 learning objectives were evaluated using a Likert scale from 1 to 4 (4 = Strongly agree). Out of the maximum score of 28, Spring 2019 had a mean ± SEM score of 26.91 ± 0.39, Fall 2019 was 24.75 ± 1.01, Spring 2020 was 24.8 ± 0.83, and Spring 2021 was 24.9 ± 0.90. There were no significant differences between sessions.

**Table 2:** Students’ perception of achieving program learning objectives.

Learning objective no.	Learning objective	Students’ perception^a^
1a	This training program helped me to obtain experience in team-based practice.	3.76 ± 0.06
1b	This training program helped me to obtain experience in leading an inter-professional medical team-for team leaders mostly	3.45 ± 0.14
2	This training program helped me become familiar with the roles of other health care professionals	3.74 ± 0.06
3	This training program helped me to improve clinical skills including but not limited to physical exam, effective communication, and promoting behavioral modification	3.49 ± 0.09
4a	This training program helped me to understand the basic concepts of managing patients with chronic pain	3.62 ± 0.08
4b	This training program helped me to understand the complexity of managing patients with chronic pain	3.70 ± 0.07
5	This training program helped me to review basic science knowledge related to pain including but not limited to relevant knowledge in the anatomy, physiology, pharmacology, pathology, and biochemistry	3.55 ± 0.08

^a^ Each learning objective graded on a scale of 1–4 (4 = strongly agree, 1 = strongly disagree, 0 = N/A). Data are presented as mean ± SEM.

### 3.5. Open-ended questions

When responses to the open-ended questions (see “Methods” for questions) were analyzed, common and unique themes were identified. In response to question 1, students in all participating years commonly stated that the best parts of the program included, working in an interdisciplinary team, learning from the attending physiatrist's lectures, and having the opportunity to work directly with patients. An overwhelming majority of students agreed that the program “helped me build confidence while working in an interprofessional team” and that the attending physiatrist's lectures “provided the scientific background to help inform each discipline's understanding of chronic pain.”

In response to question 2, students similarly noted that the program should increase the amount of time students spend with patients and create a more organized schedule for team meetings. Students noted that they “would like to have been more involved in follow-up cases to see improvements in the patient” to be able to “strengthen the clinical relationship with the patient.” A topic unique to Spring and Fall 2019 was difficulty with attendance as personal scheduling would often overlap with team meetings and discussions. Unique themes from only Spring 2021 were the need for increased participation from all group members and a preference for non-Zoom activities stating that “it is difficult to stay engaged.”

In response to question 3, students commonly stated that they found it surprising how important an integrated health care system is to adequately manage complex chronic pain conditions. Students unanimously agreed that “interprofessional practice is key in providing excellent patient care” while acknowledging that it can be challenging to create a balance within teams to provide empathetic patient centered care.

Finally, question 4 revealed that students in all program sessions would like to advocate for interdisciplinary teams in clinical settings to better care for and understand chronic pain patients in their future healthcare practice. Students agreed that they are “more empathetic and have a much better understanding of how pain works in the body” while also being able to “work more smoothly with people on diverse teams.”

## 4. Discussion

In attempts to improve medical education regarding chronic pain, the SSIPCP was created in 2016 and has been able to successfully train students since then. Even with the addition of Zoom due to the COVID-19 pandemic, students were able to improve significantly in both chronic pain physiology knowledge and their ability to work together with others to provide patient centered care. The improved KP50 and RNPQ scores after the program indicate that students' general knowledge in chronic pain physiology improved regardless of program session, while the improved IEPS and TSS data showed that students' perception of their ability to work with other health care professionals also improved independent of the use of Zoom. Overall, program session and Zoom usage did not affect the effectiveness of the program.

It is typically assumed that to foster teamwork ability being in person with your team members is necessary to form bonds and understand workflow dynamics within a group. However, this study suggests the possibility that teamwork can be comparably nurtured in an online learning setting. Phenomenological research in 2018 comparing graduate students taking courses online vs. in-person revealed many commonalities between their teamwork experiences such as group efforts to create sustainable leadership and equal division of responsibility amongst team members. However, unique differences in each learning experience were discovered making it difficult to truly compare the two distinct modalities of learning (12). Our study confirmed that students partaking in online learning can create effective leadership while fostering an environment for sharing ideas and responsibilities. Students' open-ended comments showed that students enjoyed the experience and were able to learn from their colleagues regardless of Zoom usage. Students prior to COVID-19 voiced opinions regarding absent teammates at meetings which detracted from their experience. This was not an issue for those participating online, as students were able to log into a meeting from any location, increasing freedom and convenience for students. Further, students in all sessions stated that the SSIPCP met al.l its learning objectives while upholding its strengths regardless of Zoom usage. However, despite their improvements, students seem to have a propensity towards in-person learning as this is a common experience for clinical learning. Online learning has never been the traditional method of education and participating students most likely have grown up in face-to-face learning environments making it the more comfortable modality. Students utilizing Zoom may not be able to experience the comfortability or charm that comes with face-to-face learning, but those shortcomings are made up for in terms in freedom, availability, and convenience. It should be noted that in our program, students were not required to complete chronic pain related physical exams, but only required students to interview the patients to obtain relevant histories, which may have contributed to maintaining effectiveness of Zoom sessions.

Our results are also echoed by other reports. Recent studies during the pandemic in 2021 also provided strong support for the use of online platforms in medical training regarding Opioid Overdose Prevention and Response Training. They also showed that students preferred in person activities even when online resources were just as effective (13). A study in 2020 during the COVID-19 pandemic assessing students' attitudes towards online learning revealed that students may be more opposed to online learning because of technical difficulties, distractions due to being outside a classroom, and decreased practical/demonstrative segments of learning (14). Although online learning has its negatives, Zoom usage allows for increased schedule flexibility and enrollment of students that may have difficulties with transportation. Online learning is a viable and may be more equitable option for program activities considering the fact that it seems to be as effective as in-person learning.

Not only is Zoom useful for medical education, it can also be a beneficial tool for chronic pain patients. The emotional and mental aspect of chronic pain effects patients to a great extent. It was found that long term pain management support groups were an effective way at creating healthy coping mechanisms to maintain recovery (15). Zoom can be a way for patients to connect without having to commute, which can be difficult for someone living with chronic pain. Patients can receive the emotional and social aspects of support groups without the constraints of transportation. Online video conferencing services, such as Zoom, has potential to enhance both medical education and patient care by enhancing connections between patients and medical or other health professional students during didactic and clinical skill training. Zoom or similar platforms could significantly increase health professional students' encounters with patients throughout their training with ease of access without raising the cost dramatically. These virtual student-patient encounters are particularly beneficial for students to practice conducting in-depth interviews and to learn and further understand the psychosocial aspects of chronic pain.

Notably, the results revealed an insignificant increase in RNPQ in Fall 2019 and Spring 2021 which may be due to the increased baseline scores of the participants in those years. IEPS scores also reflected a similar pattern as no significance was found in Spring 2019 and Spring 2020. This is likely because 53.7% of students in this study have had interprofessional experience while in past sessions (Spring 2016–Fall 2018) only 36.05% had prior experience (6). This is coinciding with the increased IPE programs being implemented in various health professional programs at UNE. While we are excited about this positive change in IPE, it also reminds us that necessary future modifications of the program should be considered and implemented to adapt to the continued overall improvement in IPE.

## 5. Limitations

This study only reflects feedback and surveys from the SSIPCP, which is limited by a small sample size, changing participating students each session, and lack of a control group. Health profession may also be a contributing factor towards program efficacy, and the small and varied numbers of students from each profession made accurate analysis difficult, which may limit the study's generalizability. We were also unable to follow individual students long-term to see whether the program affected their practice later on. Program modification so that it will allow long-term assessment is desired and in consideration.

## 6. Conclusion

Although students prefer in-person program activities, the SSIPCP successfully trained healthcare students in chronic pain and its management, as well as working in an IP team through Zoom. In-person activities are important for an integrated learning environment but not always “must-to-have” in students' education. This opens new avenues to effectively enrich students' education, within and beyond the education in health care professional fields, particularly programs that are traditionally taught in-person only. Zoom and other virtual formats allow us to use various online resources more effectively to make the program more versatile, equitable, and convenient while effectively providing students with a fruitful experience. With this current experience, we are inspired to re-design our program and take full advantage of many new features of virtual teaching/learning that have been discovered by educators around the world during the COVID-19 pandemic period. This includes goals to make our program more flexible while engaging, enable more participants, and include more patient interactions in the near future.

## Data Availability

The raw data supporting the conclusions of this article will be made available by the authors, without undue reservation.

## References

[1] Dahlhamer J Lucas J Zelaya C. (2018). Prevalence of chronic pain and high-impact chronic pain among adults — united States, 2016. MMWR Morb Mortal Wkly Rep 2018;67:1001–6. 10.15585/mmwr.mm6736a2 30212442 PMC6146950

[2] Gaskin DJ Richard P. The economic costs of pain in the United States. J Pain. (2012) 13(8):715–24. 10.1016/j.jpain.2012.03.009 22607834

[3] Mezai L Murinson BB, Johns Hopkins Pain Curriculum Development Team. (2011). Pain education in North American medical schools. J Pain. 12(12):1199–208. 10.1016/j.jpain.2011.06.006 21945594 PMC13235945

[4] Rice K Ryu JE Whitehead C Katz J Webster F. Medical Trainees’ experiences of treating people with chronic pain: a lost opportunity for medical education. Acad Med. (2018) 93(5):775–80. 10.1097/ACM.0000000000002053 29140917 PMC5929494

[5] Malon J Shah P Koh W Cattabriga G Li E Cao L. Characterizing the demographics of chronic pain patients in the state of Maine using the Maine all payer claims database. BMC Public Health. (2018) 18:810. 10.1186/s12889-018-5673-5 29954350 PMC6022454

[6] Cao L Hull S. Effectiveness of educating health care professionals in managing chronic pain patients through a supervised student inter-professional pain clinic. Med.Sci.Educ. (2021) 31(2):479–88. 10.1007/s40670-020-01189-4 34457905 PMC8368652

[7] Zoom Video Communications, Inc. 2023 Form 10-K Annual Report. U.S. Securities and Exchange Commission. March 3, 2023.

[8] Harris JM Jr Fulginiti JV Gordon PR Elliott TE Davis BE Chabal C Kutob RM. (2008). KnowPain-50: a tool for assessing physician pain management education. Pain Med. 9(5):542–54. 10.1111/j.1526-4637.2007.00398.x 18266812

[9] Catley MJ O’Connell NE Moseley GL. How good is the neurophysiology of pain questionnaire? A rasch analysis of psychometric properties. J Pain. (2013) 14(8):818–27. 10.1016/j.jpain.2013.02.008 23651882

[10] McFadyen AK Maclaren WM Webster VS. The interdisciplinary education perception scale (IEPS): an alternative remodeled subscale structure and its reliability. J Interprof Care. (2007) 21(4):433–43. 10.1080/13561820701352531 17654160

[11] Hepburn K Tsukuda R Fasser C. Team skills scale. (1996). In: Zeiss A Heinemann GD, editors. Team performance in health care: Assessment and development. New York: Kluwer Academic/Plenum Publishers.

[12] Saghafian M O’Neill DK. A phenomenological study of teamwork in online and face-to-face student teams. High Educ. (2018) 75:57–73. 10.1007/s10734-017-0122-4

[13] Moses TEH Moreno JL Greenwald MK Waineo E. Training medical students in opioid overdose prevention and response: comparison of in-person versus online formats. Med Educ Online. (2021) 26(1):1994906. 10.1080/10872981.2021.1994906 34727840 PMC8567883

[14] Roy H Ray K Saha S Ghosal AK. Study on Students’ perceptions for online zoom-app based flipped class sessions on anatomy organised during the lockdown period of COVID-19 epoch. J Clin of Diagn Res. (2020) 14:6. AC01-AC04. 10.7860/JCDR/2020/44869/13797.

[15] Farr M Brant H Patel R Linton MJ Ambler N Vyas S Experiences of patient-led chronic pain peer support groups after pain management programs: a qualitative study. Pain Med. (2021 Dec 11) 22(12):2884–95. 10.1093/pm/pnab189 34180996 PMC8665998

